# Chest CT Findings in a Pregnant Patient with 2019 Novel Coronavirus Disease

**DOI:** 10.4274/balkanmedj.galenos.2020.2020.3.89

**Published:** 2020-06-01

**Authors:** Xinggui Liao, Huan Yang, Junfeng Kong, Hongbing Yang

**Affiliations:** 1 Clinic of Obstetrics and Gynecology, Chongqing Three Gorges Central Hospital, Chongqing, China; 2 Chongqing University Three Gorges Hospital, Chongqing, China; 3 Clinic of Radiology, Chongqing Three Gorges Central Hospital, Chongqing, China

To the Editor,

With the ongoing outbreak of 2019 novel coronavirus (COVID-19) in December 2019, the diagnosis and treatment of this disease are critically important to clinicians. Presently, chest computed tomography (CT) and nucleic acid test are still the two most important auxiliary examinations in the COVID-19 diagnosis, according to the constantly updating diagnosis and treatment standards. The chest CT imaging of the general population with COVID-19 was described as bilateral pulmonary parenchymal ground-glass opacity (GGO) and consolidation in earlier published papers (1). A few cases about pregnant women with COVID-19 had been reported. Here, we present the characteristics of CT changes, from onset to recovery, in the lungs of a pregnant woman with COVID-19, which may help with the future diagnosis and treatment of this disease.

A 25-year-old woman who was 35 weeks and 1 day pregnant was admitted to a local hospital on February 9, 2020, presenting with a history of fatigue and mild dry cough for 3 days. She developed fever, as evidenced by an axillary temperature of 38.3°C on the same day as her hospital admittance. On examination, the routine blood test revealed a normal leukocyte count (6.67×10^9^ cells/L; reference range: 4-10×10^9^ cells/L), elevated neutrophil ratio (86.60%; reference range: 45-75%), and reduced lymphocyte count (0.71×10^9^ cells/L; reference range: 0.8-4.0×10^9^ cells/L). Although the patient had not visited Wuhan in the previous 14 days and had no history of exposure to any confirmed COVID-19 patients in local areas, the obstetrician took a throat swab to test for the presence of severe acute respiratory syndrome coronavirus-2 (SARS-COV-2). A chest radiograph was also performed, and the results revealed patchy increased density with unclear edges in the middle and upper fields of the left lung. Chest CT was not performed on that day because she was concerned about the associated radiation exposure to the fetus.

On February 11, 2020, the nucleic acid test of the patient showed a positive result. The patient was referred to our hospital. Chest CT was performed at this time, and the results revealed obvious GGO regions with indistinct borders in both lungs (Figure 1a, b). Termination of pregnancy was performed by cesarean section after admission because of fetal distress suggested by fetal heart monitoring. To investigate whether COVID-19 is possible through maternal-fetal vertical transmission, we collected the amniotic fluid, cord blood, placenta, neonatal serum, neonatal throat swab, and neonatal anal swab for the nucleic acid test, and all of which showed negative results.

The patient received antibiotic therapy (sodium piperacillin methimazole ba temple), antiviral treatment (interferon), immune enhancement (thymopentin), and uterotonic (applied after the operation). The temperature of the patient remained normal, and the symptoms included dry cough and fatigue, which disappeared on the first day after the operation. On February 16, reexamined chest CT revealed that the regions of lung infection were significantly larger than that on admission (Figure 1c, d); specifically, there was an increased range of ground-glass density patches, and partial consolidation was observed as well as a small range of bilateral pleural effusion. In response to this finding, anti-infective therapy was intensified by commencing treatment with an additional antibiotic (moxifloxacin) and glucocorticoid. The third and final chest CT, performed on February 20, 2020, showed significant reductions in the lesion area and sites (Figure 1e, f). In addition, two consecutive nucleic acid tests from throat swabs showed negative results. The patient was transferred to the rehabilitation ward for observation on February 21. Written informed consent was obtained from the patient.

SARS-CoV-2 is a novel coronavirus belonging to the β genus, and its genetic characteristics are significantly different from those of SARS-CoV and Middle East respiratory syndrome coronavirus (2). The bat is speculated to be the intermediate host of SARS-CoV-2. The main transmission routes of SARS-CoV-2 include air, droplets, aerosol, and contact, and the outstanding characteristics of the disease caused by this virus are human-to-human transmission and family aggregation (3). The main clinical manifestations of SARS-CoV-2-infected patients include fever, fatigue, cough, and other influenza-like symptoms (4). At present, CT is an important screening tool because of its high sensitivity and convenience; it can visualize the unilateral or bilateral patchy shadows or ground-glass shadows in the lungs that are characteristics of COVID-19 (5,6). Another report involving 63 COVID-19 patients described that the main CT findings included patchy/punctate GGO, patchy consolidation, and hydrothorax. As the disease progressed in these patients, the single GGO increased, enlarged, and consolidated (7). Notably, there are very few reports of pregnant women with COVID-19, and it was unknown whether the imaging characteristics and clinical process of pregnant women with COVID-19 were consistent with non-pregnant patients. In this report, we present the chest CT characteristics of a pregnant woman with COVID-19 from admission to recovery.

The patient in our case showed mild clinical symptoms, which are similar to the report by Liu et al. (8). She was confirmed to have COVID-19 based on a positive nucleic acid test and typical viral infection signs in the lungs observed by CT. The chest CT on February 11 revealed that there were multiple plaque-like dense shadows and edge frosted-glass shadows, some of which adjoined the pleura. At the time of the scan, the patient had only a mild dry cough and fatigue, with no fever or other symptoms. The above symptoms disappeared on the first day after the operation. After 5 days of treatment, reexamined chest CT (performed on February 16) revealed an increased range of GGO, the appearance of consolidation, and a small range of bilateral pleural effusion. The result indicated the presence of aggravated lesions in the lungs, which surprised the researchers. According to a previous report by Pan et al. (7), diffuse lesions and increased lung density appear when a patient’s condition worsens. However, our patient showed an overall improved condition despite having expanding lesions in her lungs. This was consistent with the previous report that the atypical clinical symptoms and the lung consolidation were common for pregnant women with COVID-19, and CT was a modality method for therapeutic effect evaluation and severity assessment (8,9).

The observations in our case suggested that the clinical symptoms of COVID-19 can be inconsistent with the CT examination results. Although we do not currently know the mechanism responsible for this presentation, findings like these must not be treated lightly given the reports showing a rapid aggravation of the patient’s condition after an obvious improvement in clinical symptoms, thought to be associated with an inflammatory storm (10). Therefore, close monitoring and comprehensive evaluation are needed when dealing with cases of COVID-19.

## Figures and Tables

**Figure 1:**
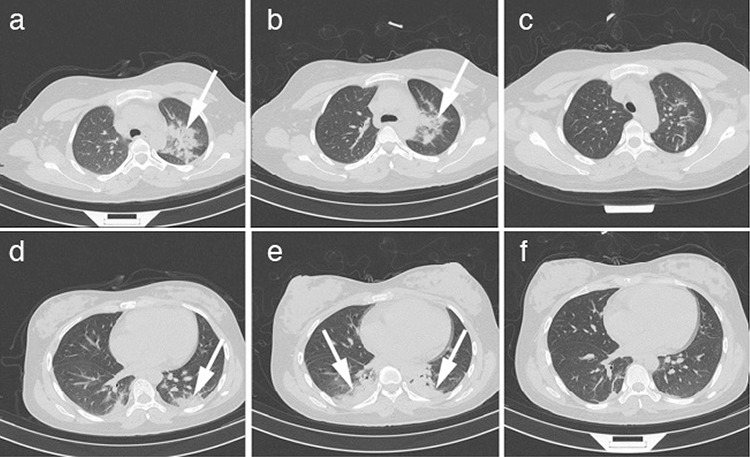
On February 11, images showed multiple plaque-like dense shadows and edge frosted glass shadows in the upper and lower lobes of bilateral lungs, part of which adjoined the pleura, and the left lung is more prominent (arrow) (a,b). On February 16, images showed a patchy shadow in the upper lobe of the left lung and narrowed slightly, but there were significant consolidation lesions in the lower lobe of both lungs (c,d). On February 20, images showed ground glass and patchy shadows of the upper and lower lobes of both lungs, which almost disappeared without obvious consolidation (e,f).
